# 

**Published:** 1800-01

**Authors:** 


### THE MEDICAL AND PHYSICAL JOURNAL;

CONTAINING

THE EARLIEST INFORMATION

ON SUBJECTS OF

*Medicine, Surgery, Pharmacy, Chemistry,*

AND

NATURAL HISTORY,

AND A CRITICAL ANALYSIS OF ALL NEW BOOKS IN THOSE DEPARTMENTS OF LITERATURE.

CONDUCTED BY

T. BRADLEY, M.D.

BY

R. BATTY, M.D.

AND BY

A. A. NOEHDEN, M.D.

171620 War

35 B

Surgeon Gen'l's Office LIBRARY. 5109, Washington, D.C.

—— Ex medicina nihil oportet putare proficisci, nisi quod ad utilitatatem corporis spectat, quoniam ejus causâ est instituta.

CICERO, *de Inventione*, Lib. I.

VOL. III.

FROM JANUARY TO JUNE, 1800.

*LONDON:*

Printed by WILLIAM THORNE, Red Lyon Court, Fleet Street,

For R. PHILLIPS, No. 71, ST. PAUL'S CHURCH-YARD.

[ Entered at Stationers Hall. ]

### ADVERTISEMENT.

*IT is consoling to the lovers of science in general, that the present times, which are eventful beyond any that can be found in the history of former ages, have not materially obstructed the progress of improvement. In the midst of the warfare and turbulence now unhappily despoiling a great part of Europe, each hostile country amicably strives to forward the cause of science, and the progress of information, which must eventually be beneficial to mankind.*

*Amongst the various efforts towards alleviating the miseries of human nature, none can have a fairer claim to the attention and thanks of the public, than those who speedily diffuse important facts and improvements in every branch of Medicine and Surgery to the world, in which* ALL *are interested; and the Conductors of the Medical and Physical Journal are happy in announcing the increasing and rapid sale of their work as the best proof of its general utility. It also gives them infinite pleasure to observe the readiness and eagerness of their numerous Correspondents, in communicating the successful or unsuccessful events resulting from their attempts to improve the method of cure of various diseases hitherto accounted incurable. In this point of view, the Medical and Physical Journal must immediately appear the most useful and ready vehicle for such facts and important pieces of information, as cannot well be published in the form of a pamphlet, but which yet are of infinite consequence to the Medical World. The change lately adopted in the distribution and arrangement of this Journal, it is presumed, has not a little increased its value and from the quantity and variety of communications already sent them, the Editors, with satisfaction, promise their readers a constant succession of useful and curious subjects for their gratification.*

NEW MEDICAL PUBLICATIONS IN DECEMBER.

AN Effay on the Prefervation of Shipwrecked Mariners, in Anfwer to the Prize-queftions propofed by the Royal Humane Society. By A. FOTHERGILL, M. D. F. R. S. &c. 8vo. 61 pp. Price 2s. 6d. *London*, Johnfon.

An Effay on the Means of leffening the Effects of Fire on the Human Body. By JAMES EARLE, Efq. F. R. S. &c. &c. 8vo. 44 pp. *London*, Johnfon.

The Art of maintaining feeble Life, and of prolonging it in incurable Difeafes. Tranflated from the German of CHRISTIAN AUG. STRUVE, M. D. 8vo. *London*, Murray and Highley.

Obferva-

Obfervations on the Bilious Fever of 1797-8-9. By RICHARD PEARSON, M. D. 1s. 6d. *London,* Baldwin.

The Firft Part of an Effay on the Anti-venereal Effects of Nitrous Acid, oxygenated Muriate of Potafh, and feveral analogous Remedies, which have been lately propofed as fubftitutes for Mercury. Likewife, the Second Part of this Effay, containing additional Evidence, with critical and practical Remarks on the Anti-venereal Remedies; and an Anfwer to fome Objections made againft the former Part. By WILLIAM BLAIR, A. M. F. R. S. *London,* H. D. Symonds.

---

### NEW PUBLICATIONS IN GERMANY.

*Phyfiologie philofophifch bearbeitet,* i. e. Phyfiology arranged upon Philofophic Principles. By CHARLES CHRISTIAN ERHARD SCHMID, Profeffor of Divinity at Jena. Vol. I. 1798. xxxiv. and 362 pp. Vol. II. 1799. viii. and 670 pp. 8vo. (Price 2 rixdoll. 18 gr. or 11s. in fheets.) *Jena,* in the Univerfity Shop.

*Handbuch der theoretifchen und praktifchen Chemie,* i. e. A Manual of the Theory and Practice of Chemiftry. By J. F. A. GOETTLING, Prof. in the Univerfity of Jena, and Member of feveral learned Societies. Two Volumes; together 1089 pp. 8vo. 1799. (Price 3 rixdoll. 4 gr. or about 13s.) *Jena,* in the Univerfity Shop.

SAM. THOM. SOEMMERING, *Icones embryonum humanorum,* 10 pp. royal folio, 1799. (Price 6 rixdoll. or 1l. 4s.) Varrentrapp and Venner. Frankfurt on the Maine.

*Filicum genera et species recentiori methodo accommodatæ analytice defcriptæ, a* JOANNE HEDWIG, M. D. ac Profeffore Botanices, &c. *Iconibus ad naturam pictis illuftratæ, a* ROMANO ADOLPHO, *filio,* Phil. et Med. Doct. 1799.—Five fheets and a half Text, and fix coloured plates, folio, (price 3 rixdoll. or about 12s.) *Leipzig.* Schäfer.

*Die neueften Entdeckungen,* &c. The lateft Difcoveries and Illuftrations in Medicine, fyftematically arranged. By F. L. AUGUSTIN, M. D. Phyfician at Berlin, &c. Vol. I. for 1798, 8vo. 564 pp. 1799. (Price 1 rixdoll. 12 gr. or about 6s.) *Berlin.* Felifch.

*Unterricht in der Wundarzneykunft,* &c. Inftructions in the Art of Surgery, compofed for the ufe of Academical Lectures. By METZGER, 8vo. 472 pp. (price 1 rixd. 6 gr. or 5s.) *Konigfberg,* Hartung.

*Syftem der Chirurgie,* i. e. A Syftem of Surgery, by ARNEMANN, with plates, Part I. Sect. I. 8vo. 336 pp. (price 1 rixd. or 4s.) *Göttingen. Vandenhök and Ruprecht.*

---

### TO CORRESPONDENTS.

*Beside those which appear in the present Number, we acknowledge the receipt of Communications from Dr. Dyce and Dr. Kinglake; Mr. Twitch, Mr. Docker, and Mr. Whyte.*

